# A Treatise on the Practice of Medicine

**Published:** 1858-11

**Authors:** 


ART. VI.—*A Treatise on the Practice of Medicine.* By GEORGE B. WOOD, M.D., Professor of the Theory and Practice of Medicine in the University of Pennsylvania, etc. etc. Fifth edition. Two volumes, 8vo. pp. 888–904. Philadelphia: J. B. Lippincott & Co., 1858.

WE hail with pleasure the appearance of the fifth edition of the above work, which, under careful revisions and large additions, and the introduction of a few new subjects, embodies all the practical points that are necessary to bring it up to the present state of our knowledge upon all the topics discussed in its comprehensive scope. Going hand in hand with that of Dr. Watson, we do not hesitate to pronounce the treatise of Dr. Wood by far the most reliable and elaborate on the Practice of Medicine that has ever appeared in this country, and one which is equalled by few in any language. It is too well known to our readers to require any commendation at our hands; being referred to by every well-informed physician and student. The mere fact that the demand has called forth five editions in a little less than eleven years, and that the work has been used as a text-book in London and Edinburgh, is the highest praise that could be bestowed upon it. Dr. Wood is an accomplished writer, and an accurate delineator of disease, and has succeeded in acquiring a reputation as an author and a teacher which any man might envy.

